# Live-born children after assisted reproduction in women with type 1 diabetes and type 2 diabetes: a nationwide cohort study

**DOI:** 10.1007/s00125-020-05193-6

**Published:** 2020-06-16

**Authors:** Michael Due Larsen, Dorte Møller Jensen, Jens Fedder, Line Riis Jølving, Bente Mertz Nørgård

**Affiliations:** 1 Department of Clinical and Molecular Medicine, Faculty of Medicine and Health Sciences, Norwegian University of Science and Technology, Erling Skjalgssons gt. 1, Laboratoriesenteret, 5. etasje, 7491 Trondheim, Norway; 2 Center for Clinical Epidemiology, Odense University Hospital, Odense, Denmark; 3 Steno Diabetes Center Odense, Odense University Hospital, Odense, Denmark; 4 Department of Gynecology and Obstetrics, Odense University Hospital, Odense, Denmark; 5 Department D, Centre of Andrology and Fertility Clinic, Odense University Hospital, Odense, Denmark; 6 Department of Clinical Research, University of Southern Denmark, Odense, Denmark; 7 Research Unit of Clinical Epidemiology, Department of Clinical Research, University of Southern Denmark, Odense, Denmark

**Keywords:** Assisted reproductive technology, Body mass index, Cohort study, Denmark, Embryo transfer, Epidemiology, In vitro fertilisation, Infertility, Type 1 diabetes, Type 2 diabetes

## Abstract

**Aims/hypothesis:**

Type 1 and type 2 diabetes are among the most prevalent chronic diseases in women in the fertile years and women with diabetes may experience several reproductive issues. We aimed to examine the chance of biochemical pregnancy, clinical pregnancy and live birth after assisted reproductive technology (ART) treatment in women with type 1 and type 2 diabetes and whether obesity per se influenced the results.

**Methods:**

This nationwide register-based cohort study is based on the Danish ART Registry comprising 594 women with either type 1 diabetes or type 2 diabetes from 2006 to 2017.

**Results:**

Relative to women without diabetes, the adjusted OR (95% CI) of a live birth per embryo transfer was 0.50 (0.36, 0.71) in women with type 2 diabetes and 1.10 (0.86, 1.41) in women with type 1 diabetes.

**Conclusions/interpretation:**

Our data on the efficacy of ART treatment in women with type 1 and type 2 diabetes is the first in this field. When compared with women without diabetes, women with type 1 diabetes had an equivalent chance of a live birth per embryo transfer whereas women with type 2 diabetes had a reduced chance. The findings in women with type 2 diabetes did not seem to be driven by obesity per se as the same pattern was seen in both normal-weight and obese women.

**Figure:**
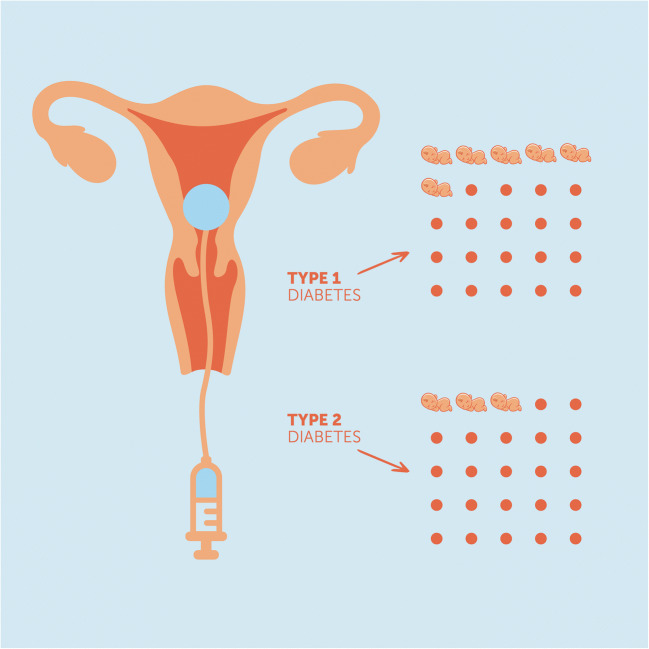
Graphical abstract

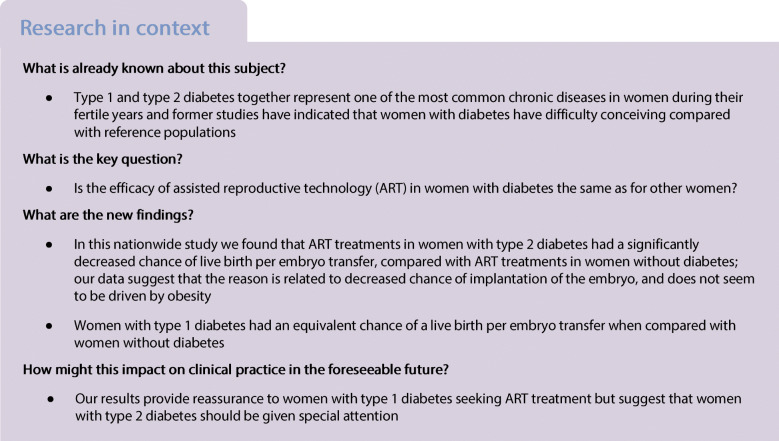



## Introduction

Type 1 and type 2 diabetes are among the most prevalent chronic diseases in women in their fertile years [1]. The state of autoimmunity is well known in type 1 diabetes [2, 3], and women with type 1 diabetes may experience several reproductive problems. Observational studies have shown an increased risk of anencephaly, microcephaly, congenital heart disease, and caudal regression, and optimising glycaemic control prior to conception is found to be associated with the lowest risk of congenital anomalies and stillbirth [4]. In addition, women with type 1 diabetes have fewer offspring than other women even though the levels of involuntary infertility has been observed to be similar to healthy women [5, 6]. In type 1 diabetes, hyperglycaemia due to insulin deficiency and hyperinsulinaemia due to exogenous insulin administration can induce hypogonadism, polycystic ovarian morphology and hyperandrogenism, resulting in decreased fertility [7]. Type 2 diabetes is associated with metabolic traits like polycystic ovary syndrome, obesity, insulin resistance and chronic inflammation. In general, both type 1 and type 2 diabetes are associated with a number of reproductive problems including reduced fertility, early and late pregnancy loss, congenital malformations, vascular stress, hypertensive disorders in pregnancy, preterm labour and infections [8–14]. These conditions are more prevalent in obese women compared with normal-weight women even in the absence of diabetes [15, 16].

Assisted reproductive technology (ART) treatment is a relevant intervention in women who cannot conceive naturally. However, recently published studies have suggested that women with rheumatoid arthritis, Crohn’s disease, ulcerative colitis and hyperthyroidism have a decreased chance of producing a live-born child after ART treatment compared with women without these conditions [17–20]. The studies also indicate that the decreased chance of a live birth might be due to failure to achieve a biochemical pregnancy and not a failure to carry the pregnancy to term. These diseases have an autoimmune aetiology, although the underlying mechanisms and the role of autoimmunity and/or chronic inflammation has yet to be examined. We aimed to examine the efficacy of ART treatment in a large group of women with type 1 and type 2 diabetes, representing women with underlying autoimmunity and chronic inflammation, respectively. Based on nationwide Danish data we thus examined the chance of biochemical pregnancy, clinical pregnancy and live birth after embryo transfer. Further, we aimed to study whether obesity per se influenced the results.

## Methods

Based on Danish health registries, this nationwide cohort study comprised all embryo transfers in Denmark from 2006 to December 2017, including follow-up on childbirths until the end of 2018. All citizens in Denmark (approximately 5.7 million inhabitants, >90% of European descent) have a unique civil registration number [21]. This number is assigned to all residents at birth and is used across all Danish health registries for valid record linkage on an individual level. We obtained the following data: (1) data related to ART procedures and cause of infertility and pregnancy from the Danish ART Registry [22, 23]; (2) data on exposure and comorbid diseases from The Danish National Patient Registry (DNPR) [24]; (3) data on the outcome of infertility treatment (live birth) from the Danish Medical Birth Registry (DMBR) [25]; and (4) data on death and immigration from The Civil Registration System [21].

### Setting and study population

Denmark has a uniform organised healthcare system and there is equal and free access to the tax-supported healthcare services, including ART treatment. Couples with fertility problems, and single women, are offered up to three in vitro fertilisation (IVF)/intracytoplasmic sperm injection (ICSI) treatment cycles with fresh embryos, and an unlimited number of frozen embryo transfers, if the woman’s age does not exceed 41 years (46 years in the private sector).

The study population comprised all women registered in the ART registry with at least one embryo transfer during the study period. The Danish ART Registry contains all treatment cycles performed in both public and private clinics [22, 23]. In this study, the term ART treatment refers to IVF (with or without fertilisation with ICSI) and transfer of frozen–thawed (frozen embryo replacement [FER]) embryos.

### Exposed and unexposed cohort

The exposed cohorts of embryo transfers in women with type 1 and type 2 diabetes were identified using the DNPR. The DNPR records all discharges from Danish hospitals since 1977 and all outpatient visits since 1994 [24]. Information in the DNPR includes details of the hospital, department, dates of admission and discharge, procedures performed and discharge diagnoses based on the International Classification of Diseases: ICD-8 before 1994 (http://www.wolfbane.com/icd/icd8.htm); ICD-10 from 1994 onwards (http://apps.who.int/classifications/icd10/browse/2016/en); ICD-9 was never used in Denmark. Diabetes coding in the DNPR has changed over time, with a single ICD-8 code of 250 in the period 1977–1986, and from 1987–1993 it changed to ICD-8 code 249 for type 1 diabetes and ICD-8 code 250 for type 2 diabetes. From 1994 onwards we used ICD-10 E10 codes for type 1 diabetes and ICD-10 E11 codes for type 2 diabetes.

Preliminary analyses revealed that 37% of the women had registered diagnostic codes for both type 1 and type 2 diabetes in the DNPR records. To avoid misclassification of types of diabetes in our analyses we defined three exposed groups: type 1 diabetes, type 2 diabetes and uncategorised diabetes. First, we included all women who had at least two discharge diagnoses of type 1 diabetes or type 2 diabetes at any time before the date of embryo transfer. Women belonging to the type 1 diabetes cohort had never had codes for type 2 diabetes, and those belonging to the type 2 diabetes cohort had never had codes for type 1 diabetes. Women who had only one diagnosis of type 1 or type 2 diabetes before embryo transfer or who had a mixture of diabetes diagnostic codes were considered to have uncategorised diabetes. We could not distinguish the type of diabetes before 1987 and therefore we started the construction of the diabetes cohorts based on data from January 1987.

The unexposed cohort comprised all embryo transfers in women without diagnoses of diabetes before the date of embryo transfer.

### Outcomes

We examined live births within a period of 140–308 days after the date of embryo transfer. We identified the information regarding live birth after ART treatment from the DMBR. The DMBR includes information on all births in Denmark since 1 January 1973 and includes information on the mother, the child and birth-related diagnoses [25, 26]. Live birth was thus considered to be the result of the particular ART treatment if the difference between embryo transfer and birth was 20–44 weeks (corresponding to 140–308 days) [17] from the start of the last menstruation. We also estimated the chance of biochemical pregnancy and clinical pregnancy as outcomes, with the purpose of estimating the women’s ability to conceive after embryo transfer. Biochemical pregnancy outcome was based on information from the ART registry (positive test for human chorionic gonadotropin measured at 14–16 days after embryo transfer) and clinical pregnancy was based on the result of an ultrasound examination approximately 7–8 weeks after embryo transfer.

### Statistical analyses

Contingency tables were constructed for the main study variables according to the three exposed cohorts and the unexposed cohort. Because each woman could have several ART treatments during the study period, the observation unit was the embryo transfer. We used multilevel logistic regression analyses to compute the crude and the adjusted RR estimates as ORs with 95% CIs for the outcomes following ART treatments in women with type 1, type 2 and uncategorised diabetes relative to women without any codes of diabetes registered. The model accounted for multiple embryo transfers in the same woman. The analyses on the chance of having a positive clinical pregnancy were based on only those women who had a positive biochemical pregnancy.

### Data on possible confounders

Data on possible confounders were obtained from the ART registry, beside data on parity and comorbid disease which were obtained from the DNPR. For classifying comorbid disease we used the Charlson comorbidity index at the time of each embryo transfer [27]. The covariates included in the regression models were selected using the modified disjunctive cause criterion [28] and the following adjustments were used in the final model: the woman’s age; cause of infertility (female factor, male factor, combined female/male factor, idiopathic infertility); calendar year of ART treatment; BMI in four categories corresponding to the WHO classification; and the partner’s smoking and alcohol intake at the time of embryo transfer. Adjusting for comorbidity in the regression model had no impact on the final estimates and was not included in final model. In subanalyses, we stratified type 2 diabetes according to BMI (normal weight or overweight/obese).

## Results

We identified 187 women with type 1 diabetes and 170 women with type 2 diabetes, undergoing 538 and 518 embryo transfers, respectively, during the study period of 2006–2017 (Table 1). We found 237 (40%) women whose diabetes we could not categorise as either type 1 or type 2 diabetes, using the DNPR. The unexposed cohort compromised a total of 127,599 embryo transfers in 42,688 women without a history of diabetes before embryo transfer. The median age of the women and their partners was similar in the exposed and unexposed cohorts. Women with type 2 diabetes were more likely to undergo IVF (45.4%) than ICSI (29.8%). The proportions of women with type 1 diabetes receiving IVF and ICSI was similar (36.6% and 39.2%, respectively) and comparable with those seen in women unexposed to diabetes.

**Table 1:** Descriptive characteristics of the study cohorts of ART treatments in women with a history of type 1 diabetes, type 2 diabetes or uncategorised diabetes and without a history of diabetes during the study period (1 January 2006 to 31 December 2017)

Characteristic	Exposed cohort			Unexposed cohort
	Embryo transfers in women with type 1 diabetes (*N* = 538)^a^	Embryo transfers in women with type 2 diabetes (*N* = 518)^b^	Embryo transfers in women with uncategorised diabetes (*N* = 682)^c^	Embryo transfers in women without diabetes (*N* = 127,599)^d^
Median (25th–75th percentile) age at embryo transfer, years	34 (30–38)	36 (32–38)	35 (32–38)	34 (30–38)
Median (25th–75th percentile) partner’s age at embryo transfer, years	36 (32–39)	37 (33–40)	36 (33–41)	36 (32–40)
Female/male factor infertility, *n* (%)				
Female factor	97 (18.0)	93 (18.0)	130 (19.1)	19,275 (15.1)
Male factor	168 (31.2)	112 (21.6)	191 (28.0)	40,990 (32.1)
A mixture of factors/idiopathic	273 (50.7)	313 (60.4)	361 (52.9)	67,270 (52.7)
Type of preceding treatment, *n* (%)				
IVF	192 (36.6)	229 (45.4)	271 (40.1)	47,061 (37.2)
ICSI	206 (39.2)	150 (29.8)	247 (36.6)	47,935 (37.9)
FER	127 (24.2)	125 (24.8)	157 (23.3)	31,490 (24.9)
BMI, *n* (%)				
<18.5 kg/m^2^ (underweight)	15 (3.4)	0 (0.0)	3 (0.5)	3518 (3.4)
18.5–25 kg/m^2^ (normal weight)	234 (53.3)	71 (17.7)	188 (33.8)	67,002 (64.1)
25–30 kg/m^2^ (pre-obesity)	130 (29.6)	138 (34.4)	162 (29.1)	23,990 (23.0)
30–35 kg/m^2^ (obese I)	54 (12.3)	142 (35.4)	155 (27.9)	8189 (7.8)
≥35 kg/m^2^ (obese II–III)	6 (1.4)	50 (12.5)	48 (8.6)	1796 (1.7)
Smoking at the time of embryo transfer, *n* (%)	38 (8.6)	48 (12.1)	60 (10.8)	8684 (8.4)
Alcohol consumer, *n* (%)	191 (45.4)	78 (21.3)	158 (30.0)	44,636 (44.8)
Calendar year of infertility treatment, *n* (%)				
2006–2009	189 (35.1)	199 (38.4)	258 (37.8)	38,259 (30.0)
2010–2013	161 (29.9)	165 (31.9)	253 (37.1)	42,305 (33.2)
2014–2017	188 (34.9)	154 (29.7)	171 (25.1)	47,035 (36.9)
Parity, *n* (%)				
0	197 (36.6)	162 (31.3)	269 (39.4)	49,612 (38.9)
1+	341 (63.4)	356 (68.7)	413 (60.6)	77,987 (61.1)
Comorbidity at embryo transfer, *n* (%)				
No comorbidity	431 (80.1)	419 (80.9)	559 (82.0)	113,726 (89.1)
Some comorbidity	107 (19.9)	99 (19.1)	123 (18.0)	13,873 (10.9)

^a^No. of women in the exposed type 1 diabetes cohort: 187. Percentage with missing data: age of partner 8.8%, ART treatment 2.4%, BMI 18.4%, smoking at the time of embryo transfer 18.0%, alcohol 21.7%

^b^No. of women in the exposed type 2 diabetes cohort: 170. Percentage with missing data: age of partner 7.5%, ART treatment 2.7%, BMI 22.6%, smoking at the time of embryo transfer 23.4%, alcohol 29.2%

^c^No. of women in the exposed uncategorised diabetes cohort: 237. Percentage with missing data: age of partner 5.4%, ART treatment 1.0%, BMI 18.5%, smoking at the time of embryo transfer 18.2%, alcohol 22.9%

^d^No. of women without a history of diabetes cohort: 42,688. Percentage with missing data: age of partner 6.8%, fertility factor 0.1%, ART treatment 2.4%, BMI 18.1%, smoking at the time of embryo transfer 18.9%, alcohol 22.0%

In general, women in the diabetes cohorts had a higher BMI than women in the unexposed cohort. We found that 82.3% of the women with type 2 diabetes had a BMI classified as pre-obese or obese (Table 1). In women with type 1 diabetes, 53.3% were normal weight and 64.1% of the unexposed women were classified normal weight. Smoking and alcohol consumption differed among the groups of women. Those with type 2 diabetes were more likely to smoke at the time of embryo transfers compared with the unexposed group (12.1% and 8.4%, respectively). On the contrary, 78.7% of women with type 2 diabetes were registered as having no alcohol use whereas among both women with type 1 diabetes and those unexposed to diabetes, approximately 55% did not consume alcohol.

### Live birth and biochemical and clinical pregnancy

The chances of biochemical pregnancy, and of clinical pregnancy, after each embryo transfer in women with type 1 diabetes, type 2 diabetes and uncategorised diabetes are shown in Table 2. In women with type 1 diabetes undergoing ART treatment, the chance of a biochemical pregnancy and clinical pregnancy was not decreased (adjusted OR [95% CI] 1.00 [0.79, 1.26] and 1.36 [0.81, 2.29], respectively). In women with type 2 diabetes the adjusted OR of a biochemical pregnancy after an embryo transfer was 0.57 (95% CI 0.43, 0.76) and the adjusted OR of a clinical pregnancy detected by an ultrasound examination was 0.91 (95% CI 0.50, 1.66).

**Table 2:** ORs for biochemical pregnancy and clinical pregnancy in women with type 1 diabetes, type 2 diabetes or uncategorised diabetes in the study cohorts of ART treatments during the study period (1 January 2006 to 31 December 2017)

	Embryo transfers in exposed cohorts	Embryo transfers in unexposed cohort	Crude OR (95% CI)	Adjusted OR (95% CI)^a^
Pregnancy in women with type 1 diabetes, *n* (%)				
Biochemical pregnancy (hCG)^b^				
Yes	202 (38.0)	45,251 (35.9)	1.10 (0.90, 1.35)	1.00 (0.79, 1.26)
No	330 (62.0)	80,862 (64.1)		
Clinical pregnancy (ultrasound)				
Yes	167 (83.5)	36,725 (82.1)	1.12 (0.74, 1.69)	1.36 (0.81, 2.29)
No	33 (16.5)	8006 (17.9)		
Pregnancy in women with type 2 diabetes, *n* (%)				
Biochemical pregnancy (hCG)^b^				
Yes	131 (25.5)	45,251 (35.9)	0.56 (0.44, 0.70)	0.57 (0.43, 0.76)
No	382 (74.5)	80,862 (64.1)		
Clinical pregnancy (ultrasound)				
Yes	101 (77.1)	36,725 (82.1)	0.72 (0.45, 1.15)	0.91 (0.50, 1.66)
No	30 (22.9)	8006 (17.9)		
Pregnancy in women with uncategorised diabetes, *n* (%)				
Biochemical pregnancy (hCG)^b^				
Yes	217 (32.2)	45,251 (35.9)	0.82 (0.68, 0.99)	0.98 (0.79, 1.21)
No	456 (67.8)	80,862 (64.1)		
Clinical pregnancy (ultrasound)				
Yes	181 (83.8)	36,725 (82.1)	1.13 (0.76, 1.70)	1.40 (0.87, 2.24)
No	35 (16.2)	8006 (17.9)		

^a^Adjusted for women’s age, calendar year of treatment, cause of infertility (female factor, male factor, or a mixture of factors/idiopathic), BMI, smoking at the time of embryo transfer and alcohol consumed

^b^Missing hCG data: type 1 diabetes 6 (0.01%); type 2 diabetes 5 (0.01%); uncategorised diabetes 9 (0.01%)

hCG, human chorionic gonadotropin

A total of 26.4% of the women with type 1 diabetes had a live-born child after embryo transfer, and this proportion was similar to that seen in the women in the unexposed cohort (23.9%), corresponding to a crude OR of 1.14 (95% CI 0.91, 1.43) for live birth and an adjusted OR of 1.10 (95% CI 0.86, 1.41) (Table 3). Only 13.5% of the women with type 2 diabetes had a live-born child after embryo transfer, and the chance of live birth was significantly reduced compared with the unexposed cohort (crude OR 0.46 [95% CI 0.34, 0.61] and adjusted OR 0.50 [95% CI 0.36, 0.71]). The adjusted OR for live birth in the group of women with uncategorised diabetes was 1.20 (95% CI 0.95, 1.51).

**Table 3:** The chance of live birth in women with type 1 diabetes, type 2 diabetes or uncategorised diabetes in the study cohorts of ART treatments during the study period (1 January 2006 to 31 December 2017)

	Embryo transfers in exposed cohort	Embryo transfers in unexposed cohort	Crude OR (95% CI) for live birth	Adjusted OR (95% CI) for live birth^a^
Women with type 1 diabetes, *n* (%)				
Yes	142 (26.4)	30,481 (23.9)	1.14 (0.91, 1.43)	1.10 (0.86, 1.41)
No	396 (73.6)	97,118 (76.1)		
Women with type 2 diabetes, *n* (%)				
Yes	70 (13.5)	30,481 (23.9)	0.46 (0.34, 0.61)	0.50 (0.36, 0.71)
No	448 (86.5)	97,118 (76.1)		
Women with uncategorised diabetes, *n* (%)				
Yes	157 (23.0)	30,481 (23.9)	0.92 (0.75, 1.13)	1.20 (0.95, 1.51)
No	525 (76.0)	97,118 (76.1)		

^a^Adjusted for women’s age, calendar year of treatment, cause of infertility (female factor, male factor, or a mixture of factors/idiopathic), BMI, smoking at the time of embryo transfer and alcohol consumed

The subanalyses restricted to women with type 2 diabetes were stratified according to weight (normal and overweight/obese). In women with normal weight and type 2 diabetes, we found a reduced chance for a live-born child following ART treatments (adjusted OR 0.37 [95% CI 0.16, 0.85]); overweight/obese women also had a reduced chance of a live-born child (adjusted OR 0.52 [95% CI 0.36, 0.77]) (Table 4).

**Table 4:** The chance of live birth in women with type 2 diabetes stratified according to BMI in the study cohort of ART treatments during the study period (1 January 2006 to 31 December 2017)

	Exposed cohort (embryo transfers in women with type 2 diabetes)	Unexposed cohort (embryo transfers in women without diabetes)	Crude OR (95% CI)	Adjusted OR (95% CI)^a^
Live birth in women with BMI 18.5–25 kg/m^2^ (normal weight), *n* (%)				
Yes	7 (9.9)	16,766 (25.0)	0.30 (0.13, 0.69)	0.37 (0.16, 0.85)
No	64 (90.1)	50,236 (75.0)		
Live birth in women with BMI ≥25 kg/m^2^ (pre-obesity/obese III), *n* (%)				
Yes	45 (13.6)	8042 (23.7)	0.48 (0.33, 0.68)	0.52 (0.36, 0.77)
No	285 (86.4)	25,933 (76.3)		

Missing data on BMI are not included in analysis

^a^Adjusted for women’s age, calendar year of treatment, cause of infertility (female factor, male factor, or a mixture of factors/idiopathic), smoking at the time of embryo transfer and alcohol consumed

## Discussion

In this nationwide cohort study encompassing 11 years of data on assisted reproduction, we found that women with type 1 diabetes did not have a reduced chance of a biochemical pregnancy, clinical pregnancy or a live-born child after ART, compared with other women receiving ART. In contrast, the chance of a live birth after an embryo transfer in women with type 2 diabetes was significantly decreased, compared with embryo transfers in women without diabetes. Our results also indicate that the reason for the decreased chance of a live birth could be related to inadequate implantation, as the chances of a biochemical pregnancy was also reduced whereas clinical pregnancy was not reduced. Our analyses considered a number of confounders, including differences in maternal weight. Even when we stratified our analyses according to categories of weight in women with type 2 diabetes, we obtained robust results. We thus found a significantly reduced chance of live birth in women with type 2 diabetes in both those who were normal weight and those who were overweight/obese.

This is the first study to examine the chance of a live-born child in women with type 1 and type 2 diabetes receiving ART treatment and therefore our results cannot be directly compared with those of others. Our findings on the efficacy of ART treatment in women with type 1 diabetes are reassuring. A previous case series study from 1992 by Dicker et al, including five women receiving IVF treatment, found that women with type 1 diabetes have the same chance of success as other women if optimal metabolic control is attained [29]. A larger study on ART and pregnancy outcome according to BMI by Pinborg et al found a reduced pregnancy rate per embryo transfer for obese women, although these results did not include information on diabetes [30].

Findings in women with other autoimmune diseases have suggested a reduced chance of live birth after ART treatment (adjusted OR [95% CI] for a live birth after ART was 0.78 [0.67, 0.91] in women with ulcerative colitis, 0.61 [0.47, 0.79] in women with Crohn’s disease, 0.78 [0.65, 0.92] in women with rheumatoid arthritis and 0.80 [0.69, 0.93] in women with hyperthyroidism) [18–20]. The results of the present study are reassuring for women with type 1 diabetes with an autoimmune aetiology, since the women with type 1 diabetes had the same chance of a positive outcome for ART treatment as women without diabetes. The decreased efficacy of ART treatment in women with type 2 diabetes indicates that inflammation and not autoimmunity is important and may provide a focus for further investigation. Women with undiagnosed type 2 diabetes and high glucose levels at the time of conception have a higher risk of spontaneous abortion and offspring with congenital malformations [8]. In addition, type 2 diabetes is more often associated with obesity, chronic inflammation, vascular stress, hypertensive conditions in pregnancy, preterm labour, pregnancy loss and infections [9]. Several studies have reported that pregnancy outcomes in women with type 2 diabetes are similar or worse than outcomes in women with type 1 diabetes [10, 14, 31]. These complications linked to hyperglycaemia are aggravated by obesity and excessive gestational weight gain [11]. Another major problem is that women with type 2 diabetes receive multi-pharmacological treatment outside pregnancy, primarily with oral glucose-lowering agents, glucagon-like peptide-1 analogues, antihypertensive drugs and cholesterol-lowering agents. Many of these drugs are potentially teratogenic and planning of pregnancy both regarding glycaemic control and review of medications is of major importance. Placenta studies have described changes in placental pathology, including villous immaturity, vascularisation, placental infarction and trophoblastic basement membrane thickness, in women with vs without diabetes [32]. The histology differs according to the type of maternal diabetes and may be linked to maternal metabolic dysfunction such as chronic inflammation, insulin resistance, hyperglycaemia, obesity and hypertension. Still, the underlying mechanisms are not fully understood. The findings of the present study support the hypothesis that the phenotypical characteristics of type 2 diabetes (chronic inflammation and insulin resistance) may increase the risk of a poor pregnancy outcome at all stages of fertilisation until the birth of the child.

It is evident that being overweight has an adverse impact on fertility, pregnancy and the health of children born to women who are above the recommended BMI levels [33–35]. Our stratified analysis showed that women with type 2 diabetes who were classed as pre-obese and obese (BMI ≥25 kg/m^2^) had a reduced chance of a live-born child. These findings suggest that the reduced success of ART in women with type 2 diabetes is not driven only by obesity but by diabetes per se.

Overall, this study is based on an unselected nationwide population from Danish health registries, which have high completeness and validity [24, 25]. We were able to consider and control for the most important confounders: women’s age, year of ART treatment, type of ART treatment, cause of infertility and BMI. This design allowed complete follow-up of the study cohorts and our outcomes were retrieved independently of the exposure status, thereby preventing selection bias and differential misclassification of the outcome. The quality of the DNPR data concerning the diagnosis of type 1 diabetes has been found satisfactory for epidemiological studies, as the predictive value of a diagnosis registration was 96% and the corresponding completeness was 91% [36]. We only included women in the type 1 diabetes cohort if they had at least two registered diagnoses of type 1 diabetes (and they never had a type 2 diabetes diagnosis) and only included women into the type 2 diabetes cohort if they had at least two registered diagnoses of type 2 diabetes (and they never had a type 1 diabetes diagnosis) before embryo transfer to ensure a high accurate exposure assessment (preventing misclassification of the exposure). It is thus a strength that we can construct two diabetes cohorts where we believe that the diagnoses are unambiguous; if there was any doubt about the specific type of diabetes, women were assigned into the uncategorised diabetes group. The ART registry is based on complete and valid data, and it is mandatory to report all treatment cycles and measurements on biochemical and clinical pregnancies from both public and private clinics [22, 23]. Regarding our outcome measurements on live birth, the data from the DMBR have both very high completeness and validity as all new-born children are registered in the DMBR [25, 26]. Unfortunately, the DNPR and Danish ATR registers used in this study do not include information on HbA_1c_. Thus, we cannot rule out that women with type 2 diabetes might have worse glycaemic control than women with type 1 diabetes. However, Danish women undergoing fertility treatment are routinely tested for diabetes and those with high HbA_1c_ are referred to a diabetologist or their general practitioner for treatment.

Our study also has limitations regarding unknown confounders, and residual confounding can never be ruled out in observational studies. In our main analyses, we have taken several confounders into consideration and we have no reason to believe that our main results are influenced by severe confounding. It would, however, have been useful to have complete information on BMI and to be able to do stratified analyses according to disease severity and medical treatment of diabetes. However, the lack of such detailed clinical information did not allow us to do so. In our subanalyses on the impact of women’s weight, we examined subcohorts of women with type 2 diabetes. Here, the statistical precision was low due to the number of women in the exposed cohorts and BMI information was missing for 18% of the women. Further stratification into subgroups of BMI was not possible due to the low number of women with BMI ≥25 kg/m^2^.

### Conclusion

Seeking help from ART has become commonplace and children born after ART treatment constitute 8% of the Danish national birth cohort [1, 37]. The increasing prevalence of type 2 diabetes in women during the reproductive period means that physicians can foresee treating and counselling an increasing number of women with type 2 diabetes. Hence, we need evidence on reproductive questions and the efficacy of ART treatment. Women starting the process of ART treatment expect information about their potential for a successful live birth and the advantages and disadvantages of ART treatment in general.

Our data on the efficacy of ART in women with type 1 and type 2 diabetes are the first data in this field. Women with type 1 diabetes had an equivalent chance of a live birth per embryo transfer when compared with women without diabetes whereas women with type 2 diabetes had a substantially reduced chance. The findings in women with type 2 diabetes did not seem to be driven by obesity per se as the same pattern was seen in both normal-weight and obese women. Prospective studies including characterisation of anthropometrics, glucose and lipid metabolism, treatment at the time of ART and biomarkers of chronic inflammation should be performed to confirm the findings in other settings and to address the mechanisms underlying these findings.

## Data Availability

The study is notified at the Danish Data Protection Agency under the current joint notification of the Region of Southern Denmark (ref.no. 2012-58-0018, 17/37434). According to Danish law, ethical review board approval or patient consent are not required for non-interventional register-based studies. According to Danish legislation, our approval to use these registers for the current study do not allow us to distribute or make patient data directly available to other parties. The authors of this paper have no special access privileges to the data used in this study, and other researchers may apply for access to data through an application to the Research Service at the Danish Health Data Authority.

## Abbreviations

ART: Assisted reproductive technology
DMBR: Danish Medical Birth Registry
DNPR: Danish National Patient Registry
FER: Frozen embryo replacement
ICSI: Intracytoplasmic sperm injection
IVF: In vitro fertilisation
